# Exogenous H_2_S facilitating ubiquitin aggregates clearance via autophagy attenuates type 2 diabetes-induced cardiomyopathy

**DOI:** 10.1038/cddis.2017.380

**Published:** 2017-08-10

**Authors:** Jichao Wu, Zhiliang Tian, Yu Sun, Cuicui Lu, Ning Liu, Zhaopeng Gao, Linxue Zhang, Shiyun Dong, Fan Yang, Xin Zhong, Changqing Xu, Fanghao Lu, Weihua Zhang

**Affiliations:** 1 Department of Pathophysiology, Harbin Medical University, Harbin 150086, China; 2 Department of Pediatrics, The Second Affiliated Hospital of Harbin Medical University, Harbin, China; 3 Department of Pharmacy, Shandong Provincial Hospital Affiliated to Shandong University, Jinan 250021, China; 4 Key Laboratory of Cardiovascular Medicine Research (Harbin Medical University), Ministry of Education, Harbin, China

## Abstract

Diabetic cardiomyopathy (DCM) is a serious complication of diabetes. Hydrogen sulphide (H_2_S), a newly found gaseous signalling molecule, has an important role in many regulatory functions. The purpose of this study is to investigate the effects of exogenous H_2_S on autophagy and its possible mechanism in DCM induced by type II diabetes (T2DCM). In this study, we found that sodium hydrosulphide (NaHS) attenuated the augment in left ventricular (LV) mass and increased LV volume, decreased reactive oxygen species (ROS) production and ameliorated H_2_S production in the hearts of db/db mice. NaHS facilitated autophagosome content degradation, reduced the expression of P62 (a known substrate of autophagy) and increased the expression of microtubule-associated protein 1 light chain 3 II. It also increased the expression of autophagy-related protein 7 (ATG7) and Beclin1 in db/db mouse hearts. NaHS increased the expression of Kelch-like ECH-associated protein 1 (Keap-1) and reduced the ubiquitylation level in the hearts of db/db mice. 1,4-Dithiothreitol, an inhibitor of disulphide bonds, increased the ubiquitylation level of Keap-1, suppressed the expression of Keap-1 and abolished the effects of NaHS on ubiquitin aggregate clearance and ROS production in H9C2 cells treated with high glucose and palmitate. Overall, we concluded that exogenous H_2_S promoted ubiquitin aggregate clearance via autophagy, which might exert its antioxidative effect in db/db mouse myocardia. Moreover, exogenous H_2_S increased Keap-1 expression by suppressing its ubiquitylation, which might have an important role in ubiquitin aggregate clearance via autophagy. Our findings provide new insight into the mechanisms responsible for the antioxidative effects of H_2_S in the context of T2DCM.

Diabetes mellitus has been firmly established as a major threat to human health due to its severe complications in the cardiovascular system.^1^ Diabetic cardiomyopathy (DCM) is one of the serious complications of diabetes, which greatly increases the incidence and severity of heart failure in patients with type 2 diabetes.^2^ Type 2 diabetes mellitus is defined as a protein-misfolding disease, which is characterized by misfolded and aggregated peptides and proteins.^3^ The accumulation of misfolded proteins results in a prolonged unfolded protein response, which contributes to mitochondria injury, reactive oxygen species (ROS) production and apoptosis. The prolonged unfolded protein response could also obstruct the ubiquitin–proteasome system leading to ubiquitinated proteins aggregating.^4, 5^

Autophagy is one of the protective factors for cell survival and is involved in eliminating damaged proteins and organelles, and autophagy has an important role in DCM.^6, 7^ Damaged organelles could be eliminated through autophagy, which preserves their functions and suppresses mitochondrial ROS production.^8, 9^ Meanwhile, the ubiquitin aggregates that can lead to apoptosis and ROS production are mainly cleared by autophagy.^10, 11^

Hydrogen sulphide (H_2_S), a newly found gaseous signalling molecule, has an important role in many regulatory functions, such as vasodilatation, antioxidation and smooth muscle relaxation.^12, 13^ In mammalian tissues, the biosynthesis of H_2_S is catalysed by the pyridoxal-5-phosphate-dependent enzymes, including cystathionine-*β*-synthetase, cystathionine-*γ*-lyase (CSE) and 3-mercaptopyruvate sulphurtransferase. In the cardiovascular system, H_2_S synthesis is mainly catalysed by CSE. Studies also indicate that H_2_S could promote disulphide formation between two Kelch-like ECH-associated protein 1 (Keap-1) molecules.^14^ Zhou *et al.*^15^ have demonstrated that exogenous H_2_S could prevent the development of DCM by reducing ROS production through the Keap-1-nuclear respiratory factor 2 (Nrf2) signalling pathway. Meanwhile, some evidence has revealed that Keap-1 has a pivotal role in ubiquitin aggregate clearance via autophagy in HEK293T cells.^16^ Although our previous study reported that H_2_S could attenuate the apoptosis of DCM induced by type 1 diabetes,^17^ the mechanism is not very clear between H_2_S and DCM. Our present study reports that exogenous H_2_S could protect myocardiocytes by promoting autophagy in type 2 diabetes. The effect of exogenous H_2_S might contribute to upregulating Keap-1 expression. Keap-1 could facilitate p62-mediated ubiquitin aggregate clearance via autophagy, which could be a key pathway for the protective effects of exogenous on myocardiocytes in type 2 diabetes. Therefore, we speculated that exogenous H_2_S was likely to increase the expression of Keap-1 by suppressing its ubiquitylation, which contributes to ubiquitin aggregate clearance via autophagy.

## Results

### Exogenous H_2_S improved cardiac diastolic function and H_2_S production in db/db mice

**Figure 1:**
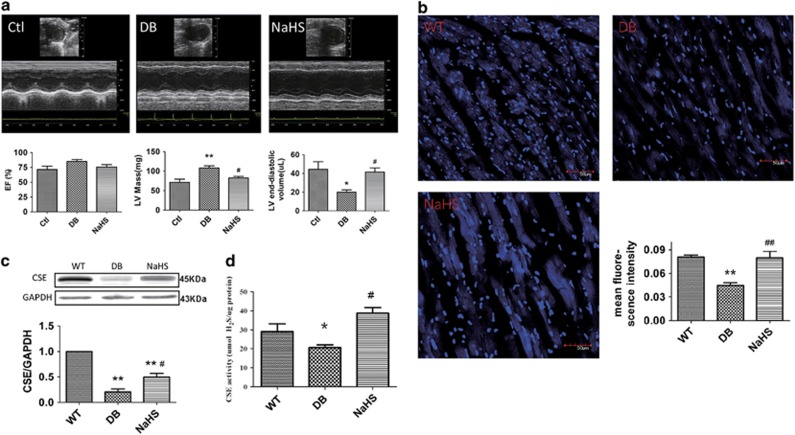
Exogenous H_2_S could protect cardiomyocytes and improve H_2_S levels in type 2 diabetes. Eight- to ten-week-old db/db mice treated with or without 100 *μ*mol/kg NaHS by intraperitoneal injection were kept on a standard chow diet for 12 weeks. (**a**) The cardiac function of mice was examined by heart echocardiography. (**b**) The content of H_2_S was detected by an H_2_S probe in mouse myocardia (blue); scale bars: 50 *μ*m. (**c**) The expression of CSE in mouse hearts was detected by western blotting. (**d**) The activity of CSE in mouse hearts was detected using CSE detection kits. Values are presented as the mean±S.D. from *n*=6 replicates. *P<0.05, **P<0.01 compared with the WT group; ^#^*P<*0.05, ^##^*P<*0.01 compared with the db/db group

The db/db mice, leptin receptor-deficient mice, were chosen as the type 2 diabetes animal model. The blood glucose levels and serum lipids were increased, and glucose tolerance was significantly decreased in db/db mice, whereas there was no effect after sodium hydrosulphide (NaHS) injection (Supplementary Figures S1a and b). To investigate the effects of exogenous H_2_S on cardiac function in db/db mice, we observed the cardiac functions of mice using echocardiography. Though the ejection fraction (EF) did not change in db/db mice, the left ventricular (LV) mass was increased, and the LV volume was decreased. These alterations were ameliorated by NaHS (Figure 1a). These results suggested that db/db mice suffering from DCM could be alleviated by exogenous H_2_S. H_2_S is an important gaseous signalling molecule. Although our previous study showed that exogenous H_2_S improved H_2_S production in a type I diabetic model,^18^ here we reveal that H_2_S concentration can elicit changes in the hearts of db/db mice. The H_2_S probe 7-azido-4-methylcoumarin (C-7Az) was used to test the H_2_S levels in mouse hearts. The results showed that H_2_S levels were decreased in the hearts of db/db mice and those levels recovered after NaHS injection (Figure 1b). We also tested the expression and activity of CSE in the hearts of db/db mice. The results showed that the expression and activity of CSE were downregulated in db/db mice, while they were elevated with the treatment of NaHS (Figures 1c and d). Furthermore, exogenous H_2_S protected cardiomyocytes against apoptosis in the hearts of db/db mice (Supplementary Figure S2).

H9C2 cells were treated with high glucose (HG) and palmitate (HG+Pal) to mimic cardiomyocytes in type 2 diabetes, and the results showed that the H_2_S levels and expression of CSE were downregulated in the HG+Pal group but were elevated with the treatment of NaHS (Supplementary Figures S3a and b). Meanwhile, the ratio of apoptotic cells was increased in the HG+Pal group, whereas it was decreased with the treatment of NaHS (Supplementary Figure S3c).

### Exogenous H_2_S attenuated ROS production in cardiomyocytes

**Figure 2:**
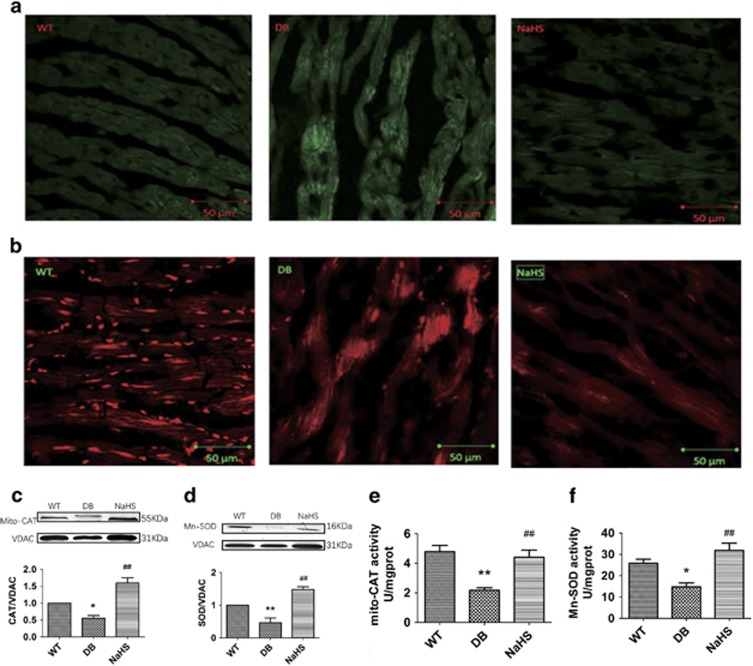
Exogenous H_2_S attenuated ROS production in db/db mouse hearts. (**a** and **b**) Cytosolic and mitochondrial ROS production was detected by DCFH stain and Mito-SOX in mouse hearts; scale bars, 50 *μ*m. (**c** and **d**) The expression of Mito-CAT and Mn-SOD in mouse hearts was detected by western blotting. (**e** and **f**) The activity of Mito-CAT and Mn-SOD in mouse hearts was detected using activity assay kits. Values are presented as the mean±S.D. from *n*=6 replicates. *P<0.05, **P<0.01 compared with the WT group; ^#^*P<*0.05, ^##^*P<*0.01 compared with the db/db group

It has been reported that oxidative stress can promote DCM.^19^ To investigate the effect of exogenous H_2_S on oxidative stress, we used 2′,7′-dichlorofluorescin diacetate (DCFH-DA) and mito-SOX staining to examine the cytosolic and mitochondrial ROS content, respectively, in mouse hearts. The results showed that the fluorescence intensity in the hearts of db/db mice was enhanced, whereas NaHS attenuated these alterations (Figures 2a and b). The expression and activity of mitochondrial catalase (Mito-CAT) and manganese-dependent superoxide dismutase (Mn-SOD) were detected to further examine the role of exogenous H_2_S on ROS production in the hearts of db/db mice. The results showed that the expression and activity of Mito-CAT and Mn-SOD, which were downregulated in the hearts of db/db mice, were upregulated by NaHS treatment (Figures 2c–f). Furthermore, exogenous H_2_S suppressed the ROS production in H9C2 cells treated with HG and palmitate (Supplementary Figure S4), which reinforced the concept that exogenous H_2_S attenuated oxidative stress in cardiomyocytes.

### Exogenous H_2_S had no significant effects on Nrf2 nuclear translocation

**Figure 3:**
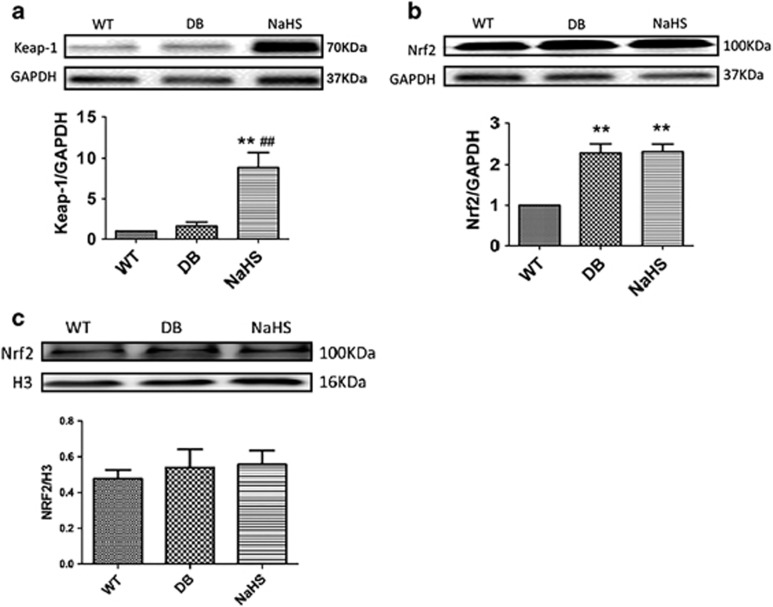
The effects of H_2_S on the Keap-1/Nrf2 pathway in db/db mouse hearts. (**a**) The expression of Keap-1 was detected by western blotting in mouse myocardia. (**b** and **c**) The expression of Nrf2 was detected by western blotting in mouse hearts and the nucleus. Values are presented as the mean±S.D. from *n*=6 replicates. **P<0.01 compared with the WT group; ^##^*P<*0.01 compared with the db/db group

It has been proven that exogenous H_2_S attenuates oxidative stress through the Keap-1/Nrf2 pathway.^19, 20, 21, 22^ Therefore, we detected the expression of Keap-1 and Nrf2. The results showed that the expression of Keap-1 was significantly upregulated by exogenous H_2_S (Figure 3a), whereas the expression of Nrf2 was upregulated in the hearts of both in db/db mice and NaHS injection mice (Figure 3b). However, the expression of Nrf2 in the nucleus showed no significant change (Figure 3c). These results imply that exogenous H_2_S had no significant effects on the Keap-1/Nrf2 pathway and that its antioxidative effects should be attributed to another mechanism.

### Exogenous H_2_S facilitated ubiquitin aggregate clearance via autophagy by suppressing ubiquitylation of Keap-1

**Figure 4:**
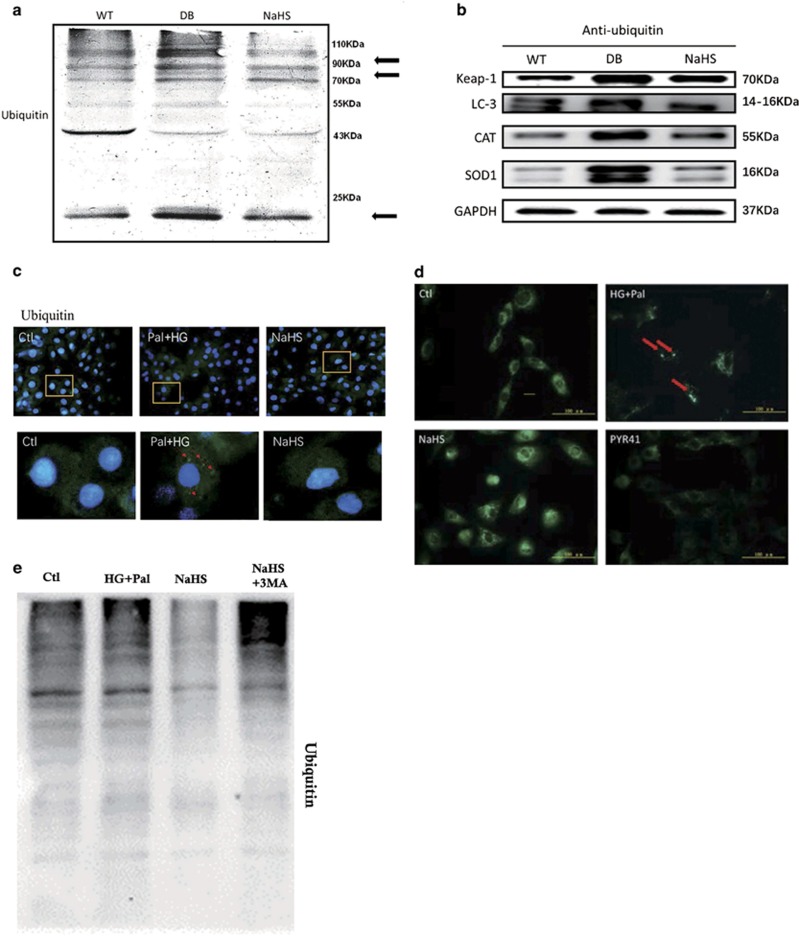
Exogenous H_2_S attenuated ubiquitylation levels. (**a**) The expression of ubiquitous protein was analysed by western blotting in mouse myocardia. (**b**) The ubiquitylation level of Keap-1, SOD and CAT in mice myocardium was detected by immunoprecipitation and western blotting. H9C2 cells were treated with HG (40 mM)+palmitate (Pal, 200 *μ*M), HG+Pal+NaHS (100 *μ*M), HG+Pal+NaHS+3MA (2 mM, an inhibitor of autophagy) and HG+Pal+PYR41 (3 *μ*M, an inhibitor of ubiquitin-activating enzyme (E1) for 48 h. (**c**) The ubiquitinated proteins in H9C2 cells were detected by an immunofluorescent assay (green); red arrows indicate ubiquitinated protein aggregates; scale bar: 100 *μ*m. (**d**) The autophagosomes were detected by MDC test in H9C2 cells (green). Red arrows indicate autophagosome accumulation; scale bar: 100 *μ*m. (**e**) The ubiquitinated protein level was detected by western blotting

To find a new explanation for the antioxidative effect of H_2_S, the effect of Keap-1 on facilitating ubiquitin aggregate clearance was considered. It has been shown that Keap-1 has a pivotal role in eliminating ubiquitin aggregates.^16^ Therefore, we detected the ubiquitylation level in mouse hearts. The results showed that the ubiquitylation levels of 90–110, 70–90 and 10 kDa proteins were increased in the hearts of db/db mice and that this alteration was attenuated with the treatment of NaHS (Figure 4a). Meanwhile, the ubiquitylation levels of Keap-1, CAT and SOD were increased in the hearts of db/db mice, while they were attenuated following NaHS treatment (Figure 4b). This might be the reason for exogenous H_2_S upregulating Keap-1 expression and suppressing ROS production. To further investigate the effect of exogenous H_2_S on ubiquitin aggregate clearance, we observed the ubiquitylation level in H9C2 cells using an immunofluorescent assay. The results showed that the ubiquitin-positive protein aggregates accumulated in the HG+Pal group, whereas NaHS promoted ubiquitin aggregate clearance (Figure 4c, red arrows). It has been reported that ubiquitin aggregates are mainly eliminated by autophagy.^23^ To determine whether the ubiquitylated proteins were the main source of autophagosomes content in the cells, PYR41 (3 *μ*M), an inhibitor of ubiquitin-activating enzyme (E1), was used to suppress the ubiquitylation. The autophagosomes aggregated in the HG+Pal group and PYR41 eliminated the autophagosome aggregates (Figure 4d, red arrows). To investigate the NaHS-facilitated ubiquitinated protein aggregate clearance via autophagy, we added 3-methyladenine (3MA) under HG+Pal+NaHS conditions and detected the ubiquitinated protein level. Our results showed that the ubiquitinated protein level was augmented in the NaHS+3MA group compared with the NaHS group and was even higher than that in the HG+Pal group (Figure 4e). These results indicate that the NaHS-facilitated ubiquitinated protein aggregate clearance occurs mainly via autophagy. The above results demonstrated that exogenous H_2_S could eliminate ubiquitin aggregates, which might be a new explanation for the antioxidative effect of H_2_S.

### Exogenous H_2_S could promote autophagy in the hearts of db/db mice and H9C2 cells

**Figure 5:**
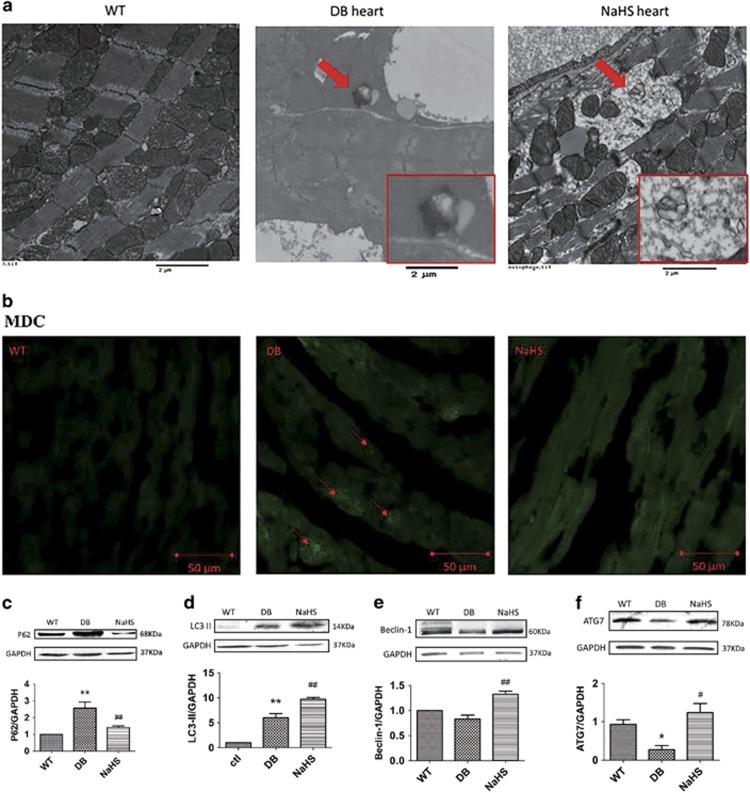
Exogenous H_2_S could promote autophagy in the hearts of db/db mice. (**a**) The ultrastructure of mouse myocardia was observed using a transmission electron microscope. The red arrow indicates an autophagosome and it was amplified in the red rectangle; scale bars: 2 *μ*m. (**b**) The autophagosome was detected using an MDC test in mouse myocardia (green). Red arrows indicate autophagosome accumulation. (**c**–**f**) The expression of P62, LC3 II, Beclin1 and ATG7 was detected by western blotting. Values are presented as the mean±S.D. from *n*=6 replicates. *P<0.05, **P<0.01 compared with the WT group; ^#^*P<*0.05, ^##^*P<*0.01 compared with the db/db group

Because ubiquitin aggregates are mainly eliminated by autophagy, and some studies have shown that autophagy is interrupted in type 2 diabetes,^24, 25^ we examined the autophagosome content in mouse hearts using a transmission electron microscope. Autophagosomes were found in the myocardiocytes of db/db mice with or without NaHS treatment. The autophagosome contents in db/db mice were not degraded and manifested as a high-density area, whereas they were degraded following NaHS treatment, manifesting as a low-density area (Figure 5a, red arrow). The monodansylcadaverine (MDC) staining showed that autophagosomes accumulated in the hearts of db/db mice, manifesting as green patches of fluorescence (Figure 5b, red arrows), which were reduced with the treatment of NaHS, and these data reinforced the concept that exogenous H_2_S facilitated autophagosome content elimination. To investigate the effect of exogenous H_2_S on the elimination of autophagosome content, we detected two autophagosomal markers, P62 and microtubule-associated protein 1 light chain 3 II (LC3 II). The results showed that the expression of P62 and LC3 II was increased in the hearts of db/db mice, whereas exogenous H_2_S reduced P62 expression but increased LC3 II expression (Figures 5c and d), which was in accordance with the disruption of autophagy and lysosome bonding.^26^ To further investigate the effects of exogenous H_2_S on autophagy, we detected the expression of two upstream factors, Beclin1 and autophagy-related protein 7 (ATG7). The results demonstrated that the expression of ATG7 was decreased in the hearts of db/db mice, and the expression of both Beclin1 and ATG7 was increased with the treatment of NaHS (Figures 5e and f), which indicated that exogenous H_2_S promoted autophagy.

**Figure 6:**
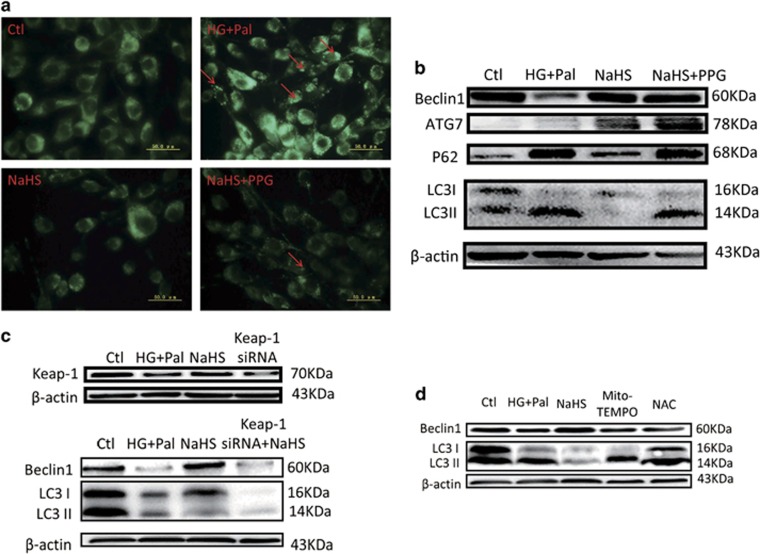
The effects of H_2_S on autophagy are attributed to Keap-1. H9C2 cells were treated with HG+Pal, HG+Pal+NaHS, HG+Pal+NaHS+PPG (10 nM, an irreversible competitive CSE inhibitor), HG+Pal+Mito-TEMPO (2 *μ*M, an inhibitor of mitochondrial ROS), HG+Pal+NAC (100 *μ*M, an inhibitor of ROS) and HG+Pal+NaHS+Keap-1 siRNA for 48 h. (**a**) The autophagosome was detected using an MDC test in H9C2 cells (green). Red arrows indicate autophagosome accumulation. (**b**) The expression of P62, ATG7, Beclin1 and LC3 II/I was detected by western blotting. (**c**) The effect of Keap-1 siRNA on autophagy was detected by western blotting. (**d**) The expression of Beclin1 and the ratio of LC3 II to LC3 I were detected by western blotting

To further demonstrate the effects of exogenous H_2_S on autophagy, we detected the autophagosomes and the expression of P62, LC3 II, ATG7 and Beclin1 in H9C2 cells. The results showed that autophagosomes accumulated in the HG+Pal group (Figure 6a, red arrows), which was attenuated with NaHS treatment. In addition, in accordance with the *in vivo* data, the expression of P62 and LC3 II was increased in the HG+Pal group, while the expression of ATG7 and Beclin1 was decreased, and these alterations were ameliorated by exogenous H_2_S (Figure 6b). dl-propargylglycine (PPG), an inhibitor of CSE, was used to investigate whether endogenous H_2_S affects autophagosome clearance under the HG+Pal+NaHS conditions. Our results showed that NaHS reduced the content of the autophagosomes in H9C2 cells treated with HG and palmitate, while the addition of PPG had no influence (Figure 6a). However, the addition of PPG increased the expression of P62 and LC3 II simultaneously, while NaHS alone decreased the expression of the two proteins (Figure 6b). These results suggested that endogenous H_2_S may have a part role in autophagosomal clearance. To investigate whether Keap-1 is the target of NaHS, the Keap-1 short interfering RNA (siRNA) was used to downregulate the expression of Keap-1. The results indicated that Keap-1 siRNA reduced the expression of Beclin1, whereas the ratio of LC3 II to LC3 I was increased (Figure 6c). To investigate whether the antioxidative effect of exogenous H_2_S played a main role in autophagy, the inhibitor of mitochondrial ROS, Mito-TEMPO, and the inhibitor of total ROS, acetylcysteine (NAC), were applied in our study. The results indicated that Mito-TEMPO and NAC reduced the expression of Beclin1 and increased the expression of LC3 II (Figure 6d), which indicated that direct inhibition of ROS production had no significant effects on autophagosomal degradation. These data suggested that the antioxidative effect of exogenous H_2_S attributed to its promotion of autophagy, which played a crucial role in ubiquitin aggregate clearance.

### Exogenous H_2_S-attenuated Keap-1 ubiquitylation possibly by promoting its disulphide formation

**Figure 7:**
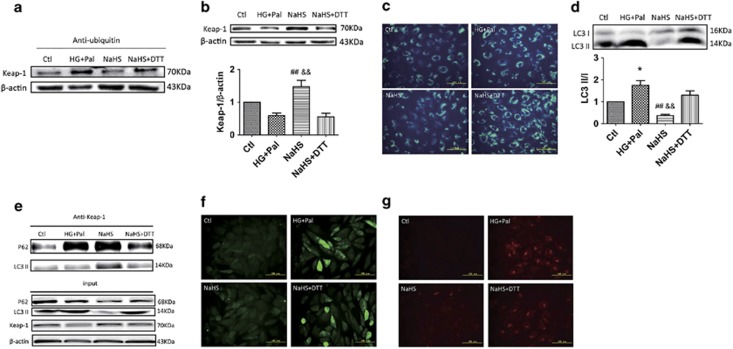
The effects of H_2_S on Keap-1 ubiquitylation might contribute to promoting disulphide formation of Keap-1. H9C2 cells were treated with HG+Pal, HG+Pal+NaHS and HG+Pal+NaHS+DTT (20 *μ*M, an inhibitor of disulphide bonds) for 48 h. (**a**) The ubiquitylation level of Keap-1 in mouse myocardia was detected by immunoprecipitation and western blotting. (**b**) The expression of Keap-1 was detected by western blotting. (**c**) The autophagosome was detected using an MDC test in H9C2 cells (green); scale bar: 100 *μ*m. (**d**) The ratio of LC3 II to LC3 I was detected by western blotting. (**e**) The interaction of Keap-1 with P62 and LC3 II was detected by immunoprecipitation and western blotting. Values are presented as the mean±S.D. from *n*=3 replicates. *P<0.05 compared with the Ctl group; ^##^*P<*0.01 compared with the HG+Pal group; ^&&^*P<*0.01 compared with the NaHS+DTT group

Some studies have reported that H_2_S could promote disulphide formation between two Keap-1 molecules.^13^ Therefore, 1,4-dithiothreitol (DTT) was used to inhibit disulphide formation. The results showed that the effects of exogenous H_2_S on Keap-1 ubiquitylation and expression were blocked with DTT treatment (Figures 7a and b). The effect of NaHS on autophagosomal elimination was also blocked with DTT treatment (Figures 7c and d). To determine whether exogenous H_2_S could promote Keap-1 interactions with P62 and LC3 II, Keap-1 was co-precipitated with P62 and LC3 II, respectively. We found that the interaction between Keap-1 and P62 was increased with or without NaHS treatment, but exogenous H_2_S also increased the interaction between Keap-1 and LC3 II. DTT blocked the effect of exogenous H_2_S on Keap-1 (Figure 7e). Furthermore, the treatment of DTT attenuated the antioxidative effect of H_2_S (Figures 7f and g), which reinforced the concept that autophagy promotion might be the reason for antioxidative effect of H_2_S.

## Discussion

The results of the current study provided new insights into the mechanisms of type 2 DCM (T2DCM) and revealed an effective protection of exogenous H_2_S in the model. Our results indicated that (i) exogenous H_2_S could attenuate ROS production and increase H_2_S production in db/db mouse hearts; (ii) exogenous H_2_S could facilitate the clearance of ubiquitin aggregates through promoting autophagy, which contributed to its antioxidative effect; and (iii) exogenous H_2_S could upregulate Keap-1 expression by suppressing its ubiquitylation.

DCM greatly increases the incidence and severity of heart failure in patients with diabetes.^2^ It has been reported that in patients with diabetes, increased LV mass^27^ and LV diastolic dysfunction, which are regarded as features for DCM, could be detected.^28^ In our study, we found that LV mass was increased and that LV volume was decreased in 20-week-old db/db mice. Hence, db/db mice were considered to be suffering cardiomyopathy on the twentieth week. NaHS attenuated these alterations, which might provide a new way to fight against T2DCM. H_2_S, as a newly found gaseous signalling molecule, has an important role in many regulatory functions.^29^ In mammalian hearts, the biosynthesis of H_2_S is mainly catalysed by CSE, and our previous study demonstrated that CSE was downregulated in type I diabetic hearts.^15^ In the current study, we also found that H_2_S production was impaired in the hearts of db/db mice, which was improved by NaHS. This inferred that H_2_S might be an important modulator in the development of T2DCM.

Increasing evidence has shown that oxidative stress can promote the development of DCM.^19^ The antioxidative stress effect of H_2_S has an important role in the functions of H_2_S.^30, 31, 32, 33^ It has been reported that H_2_S could suppress ROS production^34^ and increase SOD activity in cardiomyocytes.^35^ Our study demonstrated that exogenous H_2_S could suppress the production of ROS in the hearts of db/db mice.

However, previous studies have demonstrated that oxidative stress can interact with autophagy, and autophagy has an important role in modulating ROS production.^36, 37, 38^ Autophagy is a protective factor for cell survival, which is involved in eliminating damaged proteins and organelles, and the obstruction of autophagy could result in aggregation of injured organelles and proteins, especially injured mitochondria and ubiquitinated proteins.^5, 39, 40, 41^ Meanwhile, growing evidence has demonstrated that the development of DCM is associated with dysregulated autophagy.^42, 43^ In addition, H_2_S has its regulative roles in the autophagy process during the development and progression of many diseases such as heart failure, hepatitis and Parkinson's disease.^44, 45^ However, the effect of H_2_S on autophagy in T2DCM is only slightly illuminated. Hence, our study focused on how H_2_S regulates autophagy in the hearts of db/db mice.

Autophagy is a well-coordinated, multi-step process regulated by autophagy-related gene products and proteins such as Beclin1 and P62.^46^ In our study, we found that the autophagosome contents were not degraded in the hearts of db/db mice. Moreover, the enhanced expression of LC3 II and P62, a substrate of the autophagy-lysosomal degradation pathway, also indicated that the degradation of autophagosome contents was impaired. The upstream autophagy-related proteins Beclin1 and ATG7 were also downregulated, which might ascribe to the failed degradation of the autophagosome contents. From the above results, the obstruction of autophagy might have a pivotal role in the development of T2DCM. In addition, exogenous H_2_S facilitated autophagosome content clearance, which seemed to improve the autophagy in T2DCM. The promotive effects of exogenous H_2_S on autophagy may be an important reason for decreased ROS production.

It has been reported that ubiquitin aggregate clearance mainly depends on autophagy and the disruption of autophagy results in ubiquitin aggregate accumulation in cells.^47, 48^ We found that exogenous H_2_S reduced the ubiquitination level in the hearts of db/db mice. PYR41, an inhibitor of ubiquitination, abolished the autophagosomes formation, which indicated that ubiquitinated proteins were the main contents of autophagosome. However, how exogenous H_2_S promotion of ubiquitin aggregate clearance via autophagy still needs an explanation. Recent studies have found that Keap-1 has a crucial role in eliminating ubiquitin proteins.^16^ So Keap-1 may be a key factor for the promotive function of exogenous H_2_S on ubiquitin aggregate clearance via autophagy. We found that exogenous H_2_S upregulated the expression of Keap-1. It has also been reported that Keap-1 regulates the translocation of Nrf2, a negative regulator of ROS production, to the nucleus.^49^ However, in our study, exogenous H_2_S had no significant effects on the translocation of Nrf2 to the nucleus. A recent study by Ji and his colleague demonstrated that H_2_S suppressed diabetes-accelerated atherosclerosis via Nrf2 activation by inducing Keap-1S-sulphydration.^22^ The different findings between our and Ji’s studies are perhaps attributed to two different kinds of cells. Or rather, in a long-term high-sugar, high-fat and low-energy environment, the translocation of Nrf2 may be repressed and this might explain why H_2_S upregulates cellular antioxidants in the short-term ischaemia-reperfusion heart in a Nrf2-dependent manner^50, 51^ but had no significant effects in our study. Meanwhile, using Mito-TEMPO and NAC alone could not reduce the expression of LC3 II, which suggested that Mito-TEMPO and NAC could not promote autophagosome clearance. Therefore, we speculated that the promotion of H_2_S on autophagosomal clearance was not due to its antioxidative properties and might be the mechanism for its antioxidative properties in DCM. Therefore, the protective effect of exogenous H_2_S on Keap-1 was focused on ubiquitin aggregate clearance via autophagy. We found that in H9C2 cells, HG and palmitate increased the interaction between Keap-1 and P62 but decreased the interaction between Keap-1 and LC3 II. This indicated that the ubiquitylation level of Keap-1 was elevated, which is tethered to P62 through molecular ubiquitin, and suppressed ubiquitin aggregate clearance via autophagy. Exogenous H_2_S enhanced the interaction of both Keap-1–P62 and Keap-1–LC3 II, which reinforced that exogenous H_2_S could facilitate ubiquitin aggregate clearance via autophagy. Meanwhile, the application of Keap-1 siRNA also repressed the effect of exogenous H_2_S on autophagy. From the above, the results suggested that the effect of exogenous H_2_S on autophagy were mainly attributed to Keap-1-mediated ubiquitin aggregate clearance.

However, it is unknown how exogenous H_2_S could reduce the ubiquitylation level of Keap-1, as we found. Studies have reported that H_2_S could promote disulphide formation between two Keap-1 molecules.^22^ Therefore, we detected whether exogenous H_2_S could suppress the ubiquitylation of Keap-1 through promoting disulphide formation of Keap-1. Therefore, DTT was used to inhibit disulphide formation. We found that DTT nearly abrogated the effect of exogenous H_2_S on autophagy, ROS production and Keap-1 expression. Further, DTT inhibited the interaction of Keap-1 with P62 and LC3 II, respectively. These observations suggested that exogenous H_2_S could facilitate ubiquitin aggregate clearance via autophagy through promoting disulphide formation of Keap-1, which might contribute to ROS scavenging. However, the details of how exogenous H_2_S attenuated Keap-1 ubiquitylation needs more study.

**Figure 8:**
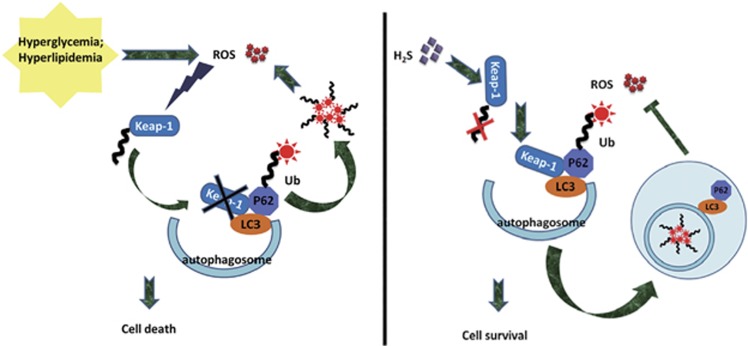
The protective effect of exogenous H_2_S on cardiomyocytes in a type 2 diabetes model. Hyperglycaemia and hyperlipidaemia induced by type 2 diabetes increase ROS production, which results in the ubiquitylation of Keap-1. As the ubiquitylation of Keap-1 was increased, the ubiquitin aggregates cannot be resolved in time. The ubiquitin aggregates accumulating in cells cause more production of ROS, which forms a vicious circle and finally induces cell death. Exogenous H_2_S could promote attenuated ubiquitylation of Keap-1, which has a protective effect on Keap-1. The increased expression of Keap-1 facilitates p62-mediated ubiquitin aggregate clearance via autophagy, which suppresses the ROS production and leads to cell survival

In summary, our results demonstrated that the cardiac impairment induced by type 2 diabetes might contribute to ubiquitin aggregate accumulation. Exogenous H_2_S could facilitate ubiquitin aggregate clearance via autophagy, which might be a new explanation for the antioxidative effect of H_2_S. The effect of exogenous H_2_S on ubiquitin aggregate clearance via autophagy might contribute to suppressing the ubiquitination of Keap-1, which upregulated the expression of Keap-1. The above evidence provides new insight into the mechanisms responsible for the antioxidative effects of H_2_S in the context of T2DCM (Figure 8). Upon description of the mechanism conferred by exogenous H_2_S protection, it is possible to provide a new avenue of therapeutic opportunities for T2DCM induced by type 2 diabetes.

## Materials and methods

### Materials

H_2_S donor NaHS, palmitate, MDC, CSE inhibitor PPG, autophagy inhibitor 3MA, disulphide bond inhibitor DTT, mitochondrial ROS inhibitor Mito-TEMPO, ROS inhibitor NAC, E1 inhibitor PYR41 and C-7Az were purchased from Sigma-Aldrich (Sigma, St. Louis, MO, USA). Mito-SOX, DCFH-DA and JC-1 were purchased from Invitrogen (Grand Island, NY, USA). SOD and CAT activity assay kits were purchased from Jiancheng Institute of Bioengineering, Nanjing, China. Keap-1 siRNA was purchased from Santa Cruz Biotechnology (Santa Cruz, CA, USA). Antibodies to cleaved caspase 3 (25546-1-AP), Bax (50599-2-lg), Bcl2 (12789-1-AP), SOD (10269-1-AP), CAT (21260-1-AP), Beclin1 (11306-1-AP), ATG7 (10088-2-AP), P62 (18420-1-AP), LC3 A/B (66139-1-lg), LC3 B (18725-1-AP), Keap-1 (10503-2-AP), ubiquitin (10201-2-AP), VDAC1 (55259-1-AP), GAPDH (10494-1-AP) and *β*-actin (66009-1-AP) were purchased from Proteintech (Rosemont, IL, USA).

### Animals

Leptin receptor-deficient (db/db) mice (8–10 weeks old) and wild-type C57BL/6 mice were purchased from the Animal Model Institute of Nanjing (Nanjing, China). The animals were housed under diurnal lighting conditions and fed standard mouse chow and water throughout the study period. All animal experiments were performed in accordance with the Guide for the Care and Use of Laboratory Animals published by the China National Institute of Health and approved by the Animal Care Committees of Harbin Medical University, China.

### Experimental groups

The animal experiment was divided into three groups. Each group included 8 mice (*n*=8). The wild-type C57BL/6 mice (8–10 weeks old) were kept on a standard chow diet for 12 weeks as control. The db/db mice (8–10 weeks old, blood glucose concentration ⩾16.7 mM) were divided into groups treated with vehicle or NaHS by intraperitoneal injection and kept on a standard chow diet for 12 weeks. The dose of NaHS was 100 *μ*mol/kg used as an effective dose in previous studies.^52, 53^

### Echocardiographic analysis of cardiac function

Cardiac functions of mice were assessed using an echocardiography system (GE VIVID7 10S, St. CT., Fairfield, USA) after 12 weeks treated. Echocardiography was performed on self-breathing mice under anaesthesia (intraperitoneal injection of 1% pentobarbital sodium at 6 ml/kg body weight). The following LV parameters were measured, including LV mass, LV end-diastolic volume and EF.

### Cell culture and treatment

Primary cultures of H9C2 rat cardiac myoblasts, purchased from the Chinese Academy of Sciences Cell Bank (Shanghai, China), were grown as monolayers at a density of 5 × 10^4^ cells/cm in Dulbecco’s modified Eagle medium and incubated at 37 °C in humidified air containing 5% CO_2_. The medium contained 10% calf serum, 100 units/ml penicillin and 100 *μ*g/ml streptomycin. Two days after being seeded, the cultured H9C2 were randomly divided into the following seven groups: control (low glucose, 5.5 mM), HG (40 mM)+palmitate (Pal, 200 *μ*M), HG+Pal+NaHS (100 *μ*M), HG+Pal+NaSH+PPG (10 nM, an irreversible competitive CSE inhibitor), HG+Pal+NaSH+3MA (2 mM, an inhibitor of autophagy), HG+Pal+NaHS+DTT (20 *μ*M, an inhibitor of disulphide bond), HG+Pal+Mito-TEMPO (2 *μ*M, an inhibitor of mitochondrial ROS), HG+Pal+NAC (100 *μ*M, an inhibitor of ROS) and HG+Pal+PYR41 (3 *μ*M, an inhibitor of ubiquitin-activating enzyme (E1)). Drugs were added directly to the culture for 48 h. H9C2 cells treated with HG and palmitate classically mimic the myocardiocytes in hyperglycaemia and hyperlipidaemia.^54^

### Detection of H_2_S in frozen sections of mouse heart using H_2_S probe C-7Az

The turn-on fluorescence response of H_2_S in RAECs was tested C-7Az, which has been demonstrated to selectively respond to H_2_S. The frozen sections of mouse hearts were incubated with 50 *μ*M C-7Az PBS for 30 min, followed by washing with PBS. Visualization of the turn-on fluorescence response of C-7Az to H_2_S in the frozen sections of mouse hearts was carried out using confocal laser scanning with the excitation of a 720 nm laser. These results confirmed that excitation fluorescence imaging could be used to detect H_2_S through the triggered fluorescence response of C-7Az.

### Mitochondrial ROS and cellular ROS level analysis

Mitochondrial ROS production was measured using Mito-SOX Red mitochondrial superoxide indicator (Invitrogen). The frozen sections of mice hearts and H9C2 cells were loaded with 5 *μ*M Mito-SOX Red at 37 °C for 15 min. Red fluorescence was measured at 583 nm following excitation at 488 nm using a Zeiss LSM 510 inverted confocal microscope (Heidenheim, Germany). Intracellular ROS levels were examined using the DCFH-DA staining method based on the conversion of non-fluorescent DCFH-DA to the highly fluorescent DCF upon intracellular oxidation by ROS. The frozen sections of mice hearts and H9C2 cells were seeded on coverslips and incubated (45 min, 37 °C, in the dark) in serum-free media containing DCFH-DA (10 *μ*M) in the presence of control, HG and NaHS. After incubation, the conversion of DCFH-DA to the fluorescent product DCF was measured using a spectrofluorometer with excitation at 484 nm and emission at 530 nm. Background fluorescence (conversion of DCFH-DA in the absence of cells) was corrected by the inclusion of parallel blanks.

### Total protein extraction from diabetic mouse hearts and H9C2 cells and western blot analysis

Diabetic mouse hearts and H9C2 cells were homogenized in 0.5 ml of RIPA buffer before being transferred into small tubes and rotated for 1 h at 4 °C. Solubilized proteins were collected after centrifugation at 3000 × *g* for 30 min. The supernatant was collected and stored at −80 °C. The protein concentration of each sample was quantified using the BCA Protein Assay kit (Beyotime, Shanghai, China). Protein lysates from each group of cells and tissues via electrophoresis were separated by SDS-PAGE and electrotransferred onto a PVDF membrane (Millipore). Polyacrylamide gels (12%) were used for protein testing. The nonspecific proteins on membranes were blocked with 5% non-fat dried milk for 2 h at room temperature. Membranes were incubated with 2 *μ*g/ml primary antibodies overnight at 4 °C. Membranes were washed and then incubated with anti-mouse/anti-rabbit IgG antibody at a 1:5000 dilution for 1 h at room temperature. The specific complex was visualized using ECL plus western blot detection system. The relative intensities of protein bands were quantified using a Bio-Rad Chemi EQ densitometer and Bio-Rad Quantity One software (Bio-Rad Laboratories, Hercules, CA, USA).

### MDC assay for visualization of autophagic vacuoles

The frozen sections of mouse hearts and H9C2 cells were incubated with 50 *μ*M MDC in PBS at 37 °C for 30 min. Autophagic vacuoles were analysed using confocal laser scanning and fluorescence microscopy (Olympus, XSZ-D2, Tokyo, Japan).

### Immunofluorescence assays

For immunofluorescent staining with anti-ubiquitin antibody, the H9C2 cells were fixed in 4% paraformaldehyde for 30 min and then permeabilized with 0.5% Triton X-100 for 30 min. Coverslips were blocked with goat serum and incubated for 1 h at 37 °C. Cells were incubated with anti-ubiquitin antibody at 4 °C overnight and washed three times with PBS, followed by incubation for 1 h with anti-rabbit IgG. Analysis and photomicrography were carried out with fluorescence microscopy.

### Immunoprecipitation

Next, 1 ml samples (2 *μ*g/*μ*l) were incubated with IgG conjugated agarose beads (50 *μ*l) and 2 *μ*l of anti-ubiquitin antibodies (following the manufacturer’s instructions) and rotating overnight at 4 °C. The IP mixture was centrifuged at 1000 r.p.m. for 30 s at 4 °C, and the supernatant was discarded. The beads were washed 3–4 times with 1 ml of RIPA lysis buffer 3 times. Then, 25 *μ*l of 5 × SDS sample buffer was added to the elutions, and the solution was heated at 95 °C for 5 min. The samples were centrifuged at 10 000 × *g* for 3 min. The supernatants were loaded onto an SDS-PAGE gel, and the supernatant was carefully transferred to a fresh, well-labelled microfuge tube and stored at −80 °C for later use. IPs was separated by SDS-PAGE, and proteins were transferred to PVDF membrane. Samples were probed with appropriate antibodies (anti-Keap-1, anti-LC3, anti-CAT and anti-SOD) for analysis.

### siRNA transfection

H9C2 cells (80% confluent) were treated according to the manufacturer’s instructions with Keap-1 siRNAs (mouse; Santa Cruz Biotechnology) for 72 h to inhibit Keap-1 expression. Transfection of H9C2 cells by siRNA was achieved using Lipofectamine 2000 (Invitrogen). In brief, Keap-1 siRNA with the transfection reagent was incubated for 20 min to form complexes, which then were added to plates containing cells and medium. The cells were incubated at 37 °C in a CO_2_ incubator for further analysis.

### Statistical analysis

Data were presented as mean±S.D. Data were first analysed using one-way ANOVA test. Tukey test was used for *post hoc* comparisons. The threshold of *P*<0.05 was designated as statistically significant for all tests. All statistical analyses were performed using Prism 5 (GraphPad, La Jolla, CA, USA).
