# Supplementary material for: Exogenous H2S facilitating ubiquitin aggregates clearance via autophagy attenuates type 2 diabetes-induced cardiomyopathy
Source: Cell Death Dis. 2017 Aug 10;8(8):e2992–. doi: 10.1038/cddis.2017.380 (PMC5596567; doi:10.1038/cddis.2017.380)
Supplement: Supplementary Information [file cddis2017380x1.docx]

**
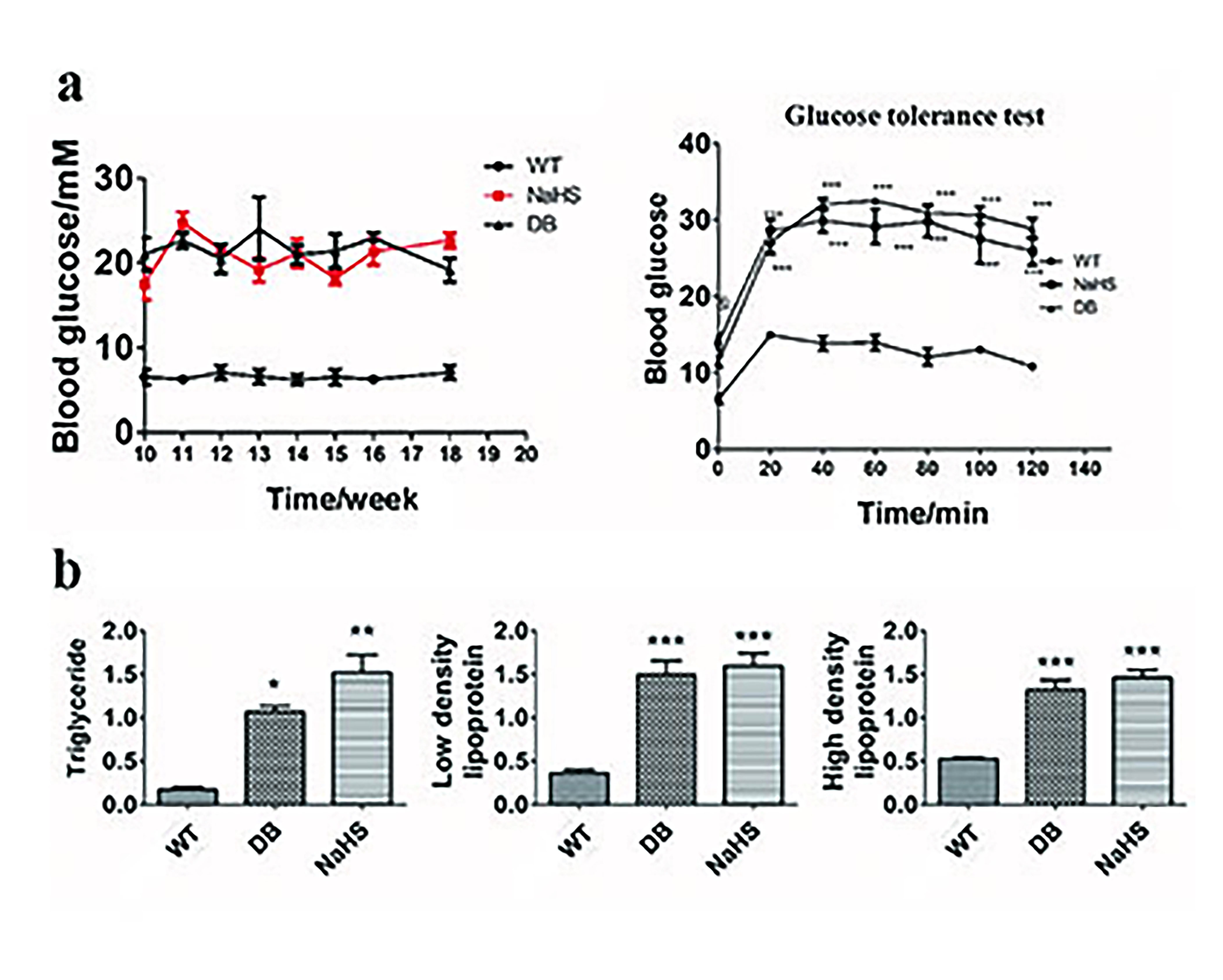
**

**Figure S1** Exogenous H_2_S had no significant effect on glucose and lipid metabolism of db/db mice. (**a**) The blood glucose concentration and tolerance of db/db mice were examined. (**b**) The content of triglyceride, low density lipoprotein and high density lipoprotein. Values are presented as mean ± S.D. from n =6 replicates. ^*^*P*＜0.05, ^**^*P*＜0.01, ^***^*P*＜0.001 compared with the WT group.

To investigate the effect of exogenous H_2_S on cardiomyocytes of db/db mice, we observed the ultrastructure using transmission electron microscope. Apoptotic cardiomyocytes were found (Figure S2a yellow arrow) in db/db mouse heart. To further examine the apoptosis of cardiomyocytes of db/db mice, TUNEL assay was applied. The result showed that the ratio of apoptotic cells was increased in db/db mice, and this measure decreased following NaHS treatment (Figure S2b). Cleaved caspase 3 (Cl-casp3) is a marker of apoptosis. The result showed that the expression of Cl-casp3 was up-regulated in the hearts of db/db mice and that expression was suppressed following NaHS treatment (Figure S2c). The expression of Bcl-2 was down-regulated in the hearts of db/db mice while the expression of Bax was up-regulated, these alterations were ameliorated by NaHS (Figure S2d and e).


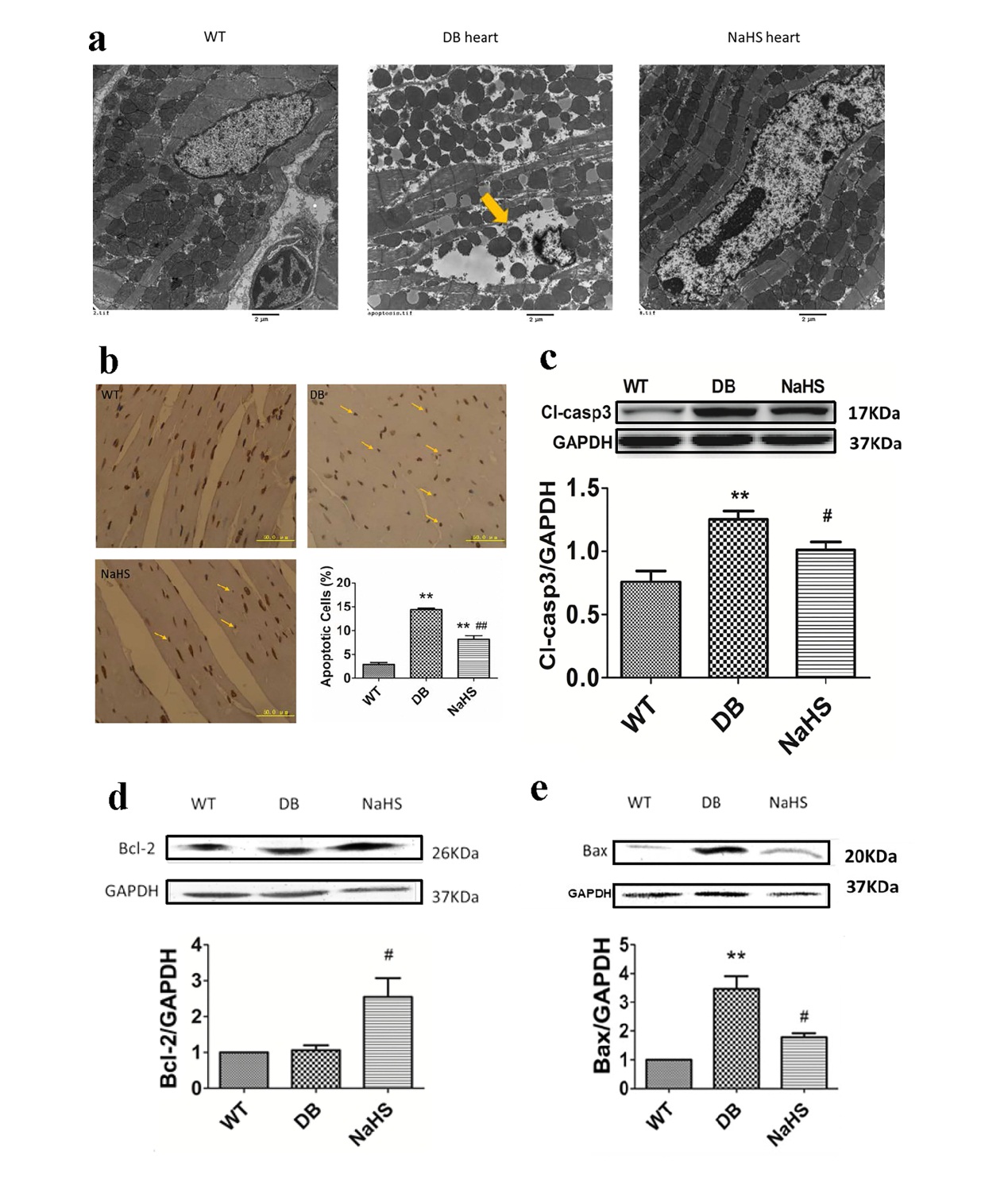


**Figure S2** Exogenous H_2_S protected cardiomyocytes against apoptosis in the hearts of db/db mice. (**a**) The ultrastructure of mice myocardium was observed using transmission electron microscope. Yellow arrow mean apoptotic cell, Scale bars, 2 μm. (**b**) Apoptosis of mice myocardium was analyzed by TUNEL staining, Scale bars, 50 μm. (**c-e**) The expression of Cl-casp3, Bcl-2 and Bax in mice heart was detected by western blot. Values are presented as mean ± S.D. from n =6 replicates. ^**^*P*＜0.01 compared with the WT group, ^#^*P*＜0.05 compared with the db/db group.

**
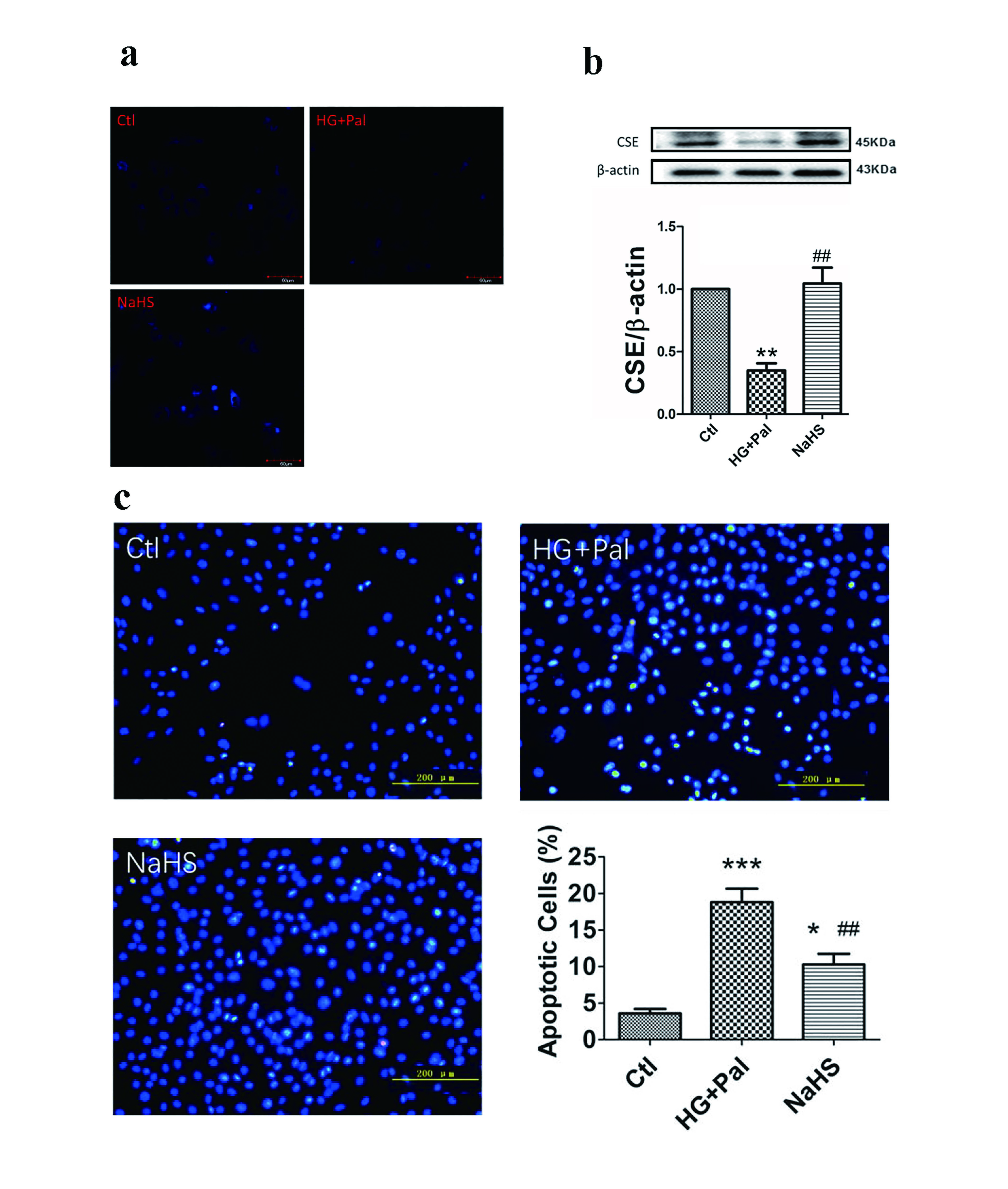
**

**Figure S3** Exogenous H_2_S attenuated apoptosis and improved H_2_S production in H9C2 cells. H9C2 cells were treated with HG+Pal and HG+Pal+NaHS for 48 hours. (**a**) The content of H_2_S was detected by H_2_S probe in H9C2 cells (blue), Scale bars, 60 μm. (**b**) The expression of CSE in H9C2 cells was detected by western blot. (**c**) The apoptotic ratio of H9C2 cells was detected by Hoechst 33342/PI, Scale bars, 200 μm. Values are presented as mean ± S.D. from n =3 replicates. ^*^*P*＜0.05, ^**^*P*＜0.01, ^***^*P*＜0.001 compared with the Ctl group; ^##^*P*＜0.01 compared with the HG+Pal group.

**
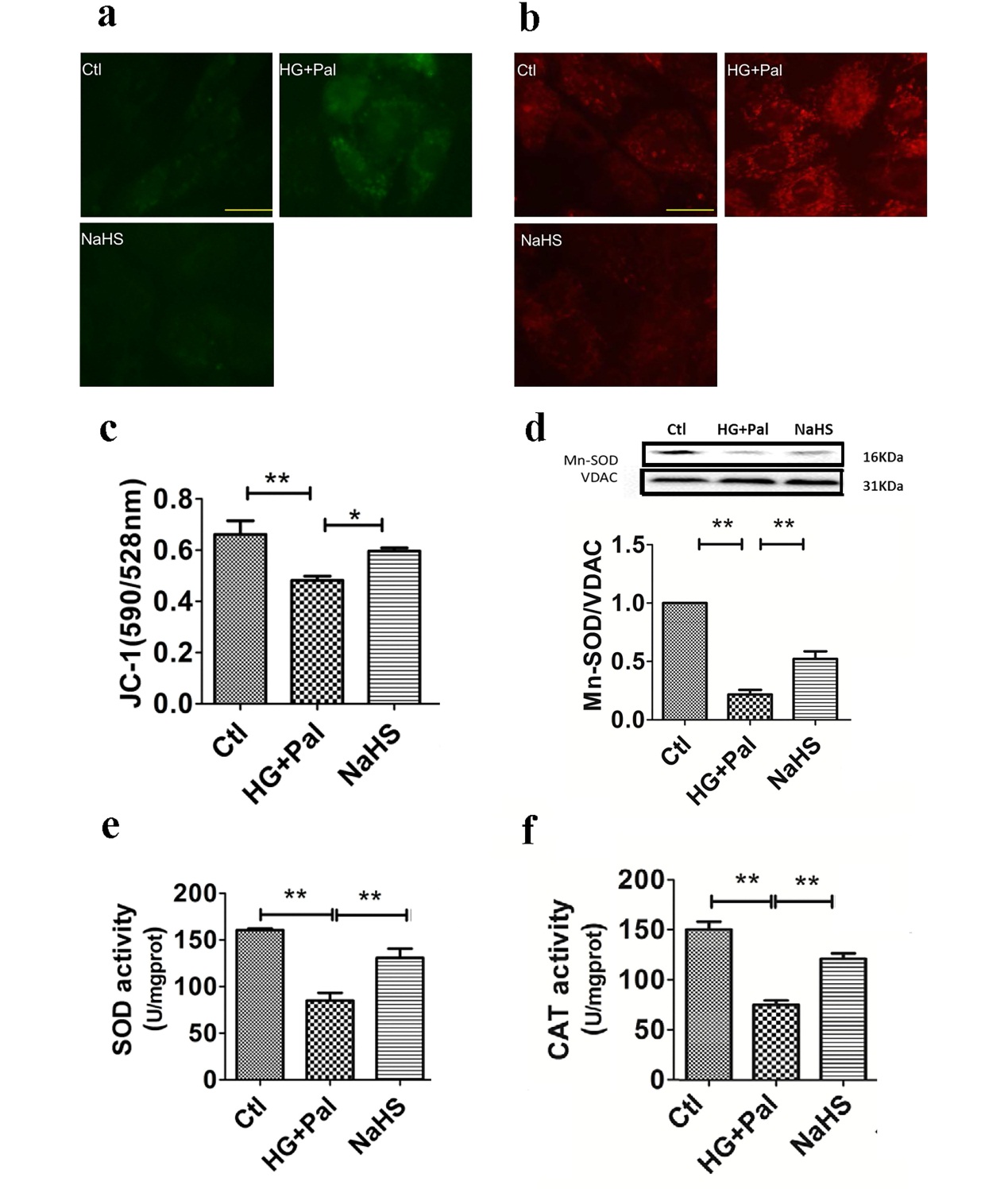
**

**Figure S4** Exogenous H_2_S attenuated ROS production in H9C2 cells. (**a** and **b**) Cytosolic and mitochondrial ROS production was detected by DCFH stain and Mito-SOX in H9C2 cells, Scale bars, 100 μm. (**c**) The mitochondrial membrane potential of H9C2 cells was detected by JC-1. (**d**) The expression of Mn-SOD in H9C2 cells was detected by western blot. (**e** and **f**) The activity of SOD and CAT in H9C2 cells was detected by activity assay kits. Values are presented as mean ± S.D. from n =3 replicates. ^*^*P*＜0.05, ^**^*P*＜0.01.

**Materials and Methods**

**Terminal deoxynucleotidyltransferase-mediated dUTP nick-end labelling (TUNEL) Staining**

TUNEL assay was used to test for apoptosis in mouse heart. The mouse hearts were fixed in 4% formalin for 48 hours. Then, the hearts were dehydrated and embedded in paraffin. Apoptotic cells were tested by a TdT DNA fragmentation detection kit (Roche, Mannheim, Germany), and the procedure was performed according to the kit’s protocol. The percentage of apoptotic cells was calculated as the ratio of the number of TUNEL-positive cells to the total number of cells.

**Hoechst 33342 / PI staining for apoptosis assay**

Cells were seeded and treated for 48 h in 24-well plates, washed three times with PBS, incubated with 20 μg·ml^-1^ Hoechst staining buffer for 15 min at 37°C in the dark and then incubated with 20 μg·ml^-1^ propidium iodide (PI) for 10 min. The percentage of apoptotic cells was observed using fluorescence microscopy.

**Measurement SOD and CAT activities**

Superoxide dismutase (SOD) and catalase (CAT) in the supernatant were measured using a spectrophotometer (Jiancheng Institute of Bioengineering, Nanjing, China). All assays were conducted according to the kit instructions.

**Analysis of mitochondrial transmembrane potential**

Changes in mitochondrial transmembrane potential were assessed using the lipophilic cationic probe 5, 5’, 6, 6’-Tetrachloro-1, 1’, 3, 3’-tetraethyl-imida-carbocyanine iodide (JC-1). In brief, H9C2 cells were seeded and treated for 24 h at 37°C. After experimentation, cells were loaded with 2 μM JC-1 (Invitrogen) at 37°C in the dark for 15 min and rinsed three times with cold PBS. Green fluorescence reflected the monomeric form of JC-1, and red fluorescence reflected the aggregate form. The cells were monitored using fluorescence microscopy.
